# SCART sequential lattice spatial fractionated radiotherapy combined with chemotherapy for recurrent ovarian cancer with bulky bone metastasis: a case report

**DOI:** 10.3389/fonc.2026.1761101

**Published:** 2026-02-04

**Authors:** Yuan Li, Liangfu Han, Weisi Yan, Jun Yang

**Affiliations:** Radiation Oncology, Foshan Fosun Chancheng Hosptal, Guangdong, Foshan, China

**Keywords:** bone metastasis, lattice, SCART, ovarian cancer, spatial fractionated radiotherapy

## Abstract

**Background:**

Bone metastasis from ovarian cancer is relatively rare, and the treatment of giant bone metastatic lesions poses significant clinical challenges. SCART (Stereotactic Centralized Ablative Radiation Therapy) and Lattice technique, as emerging spatial fractionated precision radiotherapy technologies, have shown unique advantages in treating bulky tumor lesions. This case report explores the potential immunomodulatory effects of spatial fractionated radiotherapy in enhancing tumor antigenicity and chemotherapy sensitivity in recurrent, drug-resistant, multi-line treated cancer patients.

**Case presentation:**

We report a 56-year-old female patient with ovarian cancer recurrence and multiple metastases 7 years post-surgery. CT examination revealed a big right iliac bone metastatic lesion (89×78×58mm) with surrounding soft tissue invasion. After limited response to chemotherapy and bevacizumab, the patient underwent sequential SCART, SBRT, and Lattice radiotherapy for her right iliac and surrounding invasive soft tissue metastases. Substantial local tumor regression was achieved without severe acute toxicity.

**Methods:**

The initial radiotherapy consisted of SCART followed by SBRT: STV 54 Gy/3 fractions, PGTV 15 Gy/3 fractions, then SBRT PGTV 25 Gy/5 fractions. Stage II treatment used Lattice radiotherapy: VTV 45 Gy/3 fractions and PGTV 9 Gy/3 fractions.

**Results:**

The patient’s pain symptoms were significantly relieved, with good tolerance during treatment and no severe adverse reactions. According to RECIST 1.1 evaluation criteria, the baseline GTV was 399.0cc, decreased to 308.0cc after SCART +SBRT, and further reduced to 136.1cc at 6-month follow-up MRI after Lattice radiotherapy. The diameter decreased by approximately 30.1% compared to baseline, achieving partial response.

**Conclusion:**

The combination of SCART and Lattice techniques offers a promising strategy for managing bulky tumor, particularly suitable for cases with less-responsive to conventional therapy. This technique ensures safe dose at target margins while increasing dose to “cold tumor” areas, offering new clinical options. This approach safely escalates intratumoral dose while protecting normal tissue and may enhance immunogenic and cytotoxic responses. Sequential spatial fractionation techniques appear feasible, with controllable short-term safety and favorable tolerance.

## Introduction

Ovarian cancer is one of the most common malignant tumors of the female reproductive system, ranking third in incidence among gynecological malignancies (1). Approximately 70% of patients are diagnosed at an advanced stage (2). Bone metastasis in ovarian cancer is relatively rare, with an incidence of about 2-8% (3). The treatment of giant bone metastatic lesions has always been a clinical challenge, especially when tumor volume limits conventional radiotherapy dosing.

In recent years, stereotactic radiotherapy technology has developed rapidly, further extending to non-uniform irradiation design concepts. SCART (Stereotactic Centralized Ablative Radiation Therapy), as an emerging precision radiotherapy technology, has shown unique advantages in treating large-volume tumor lesions (4). Meanwhile, the Lattice technique, as a typical representative of spatial fractionation, creates multiple small dose hotspots within the tumor, forming a “lattice-like” dose distribution that can increase irradiation dose to cold spot areas while ensuring safe dose at target margins (5). SCART and Lattice radiotherapy represent novel approaches capable of addressing the limitations of traditional treatments by delivering highly heterogeneous intratumoral dose distributions.

## Case report

### Patient information

**Figure 1:**
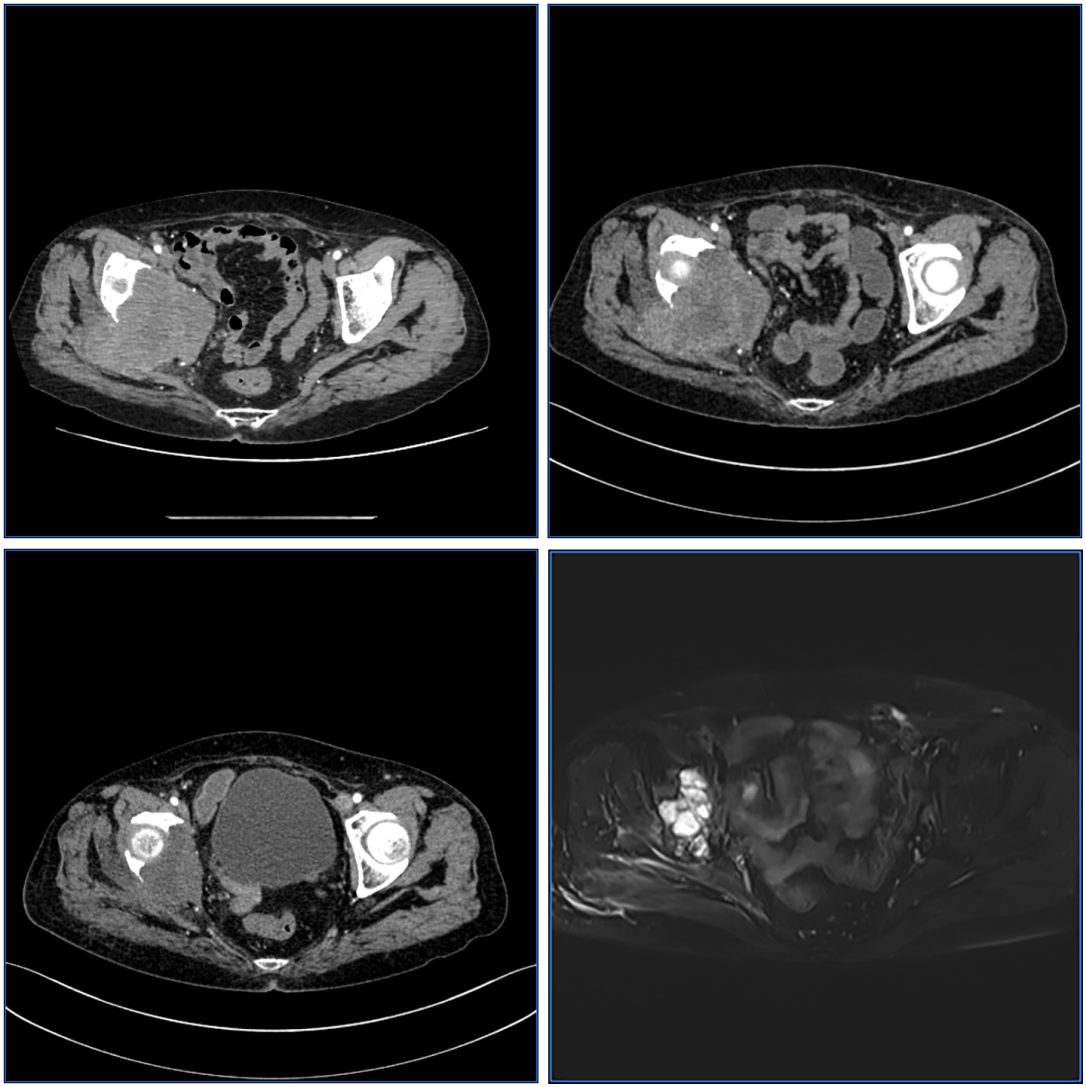
CT/MRI scans showing pelvic cross-sections at different treatment stages: “Before SCART,” “1 month after SCART,” “3 months after Lattice,” and “6 months after RT.” Each panel displays changes in tumor size and tissue density over time.

A 56-year-old woman underwent surgery for ovarian cancer in 2018, with postoperative diagnosis of “clear cell carcinoma.” Postoperative adjuvant therapy details were unknown, with regular follow-up showing stable disease. In February 2025, she presented with “worsening right hip pain for 1 month.” CT examination on February 6, 2025, showed: bone destruction in the posterior right acetabulum with soft tissue mass formation, approximately 89×78×58mm, highly suggestive of metastatic tumor; multiple pulmonary solid nodules were also found, suggesting multiple metastases (**Figure 1a**).

Right buttock mass biopsy was performed on February 10, 2025. Pathological examination showed: atypical cells arranged in tubular, cribriform, and small nest structures within proliferative fibrous tissue, considered malignant tumor metastasis. Combined with clinical history and immunohistochemical results (CA125(+), CK7(+), CK(+), PAX-8(+)), ovarian origin tumor metastasis was considered.

### Treatment process

From February 13 to April 1, 2025, the patient received albumin-bound paclitaxel + carboplatin + bevacizumab × 3 cycles. Pulmonary metastases regressed; however, the large iliac lesion remained largely unchanged.

### Radiotherapy treatment

Stage I– SCART + SBRT (April 24 – May 5, 2025) :

She was received RT for right iliac bone, acetabular bone metastasis and surrounding soft tissue invasion. STV 54 Gy/3 fractions, PGTV 15 Gy/3 fractions, followed by SBRT PGTV 25 Gy/5 fractions. Pain improved substantially (NRS 4–5 → 2). Grade 2 diarrhea occurred after radiotherapy, which resolved after symptomatic treatment.

Stage II – Lattice Radiotherapy (June 11 – June 13, 2025):

Lattice technique was continued for radiotherapy of this metastasis. Treatment plan: VTV 45 Gy/15 Gy/3 fractions, PTV 9 Gy/3 Gy/3 fractions. During treatment, patient tolerance was good without severe adverse reactions (**Tables 1**, **2**).

**Table 1:** Organ at Risk (OAR) dose constraints and actual values.

OAR	Parameter	Actual value	Threshold (Timmerman 10fractions)	Fractionation scheme
Right Femoral Head	Dmax/D10cc(10F)	43.35Gy/28.4 Gy	43.5Gy/38.0Gy	3 + 5+3 = 11 fractions (SCART+SBRT+Lattice combined)
Small Bowel	Dmax/D0.035cc(10F)	49.65Gy(0.035cc)/16.08Gy	41Gy/33.9Gy	
Colon/Rectum	Dmax	2100cGy	6500cGy	
Bladder	Dmax/D90cc	36.2Gy/6.61Gy	53Gy/48Gy	

**Table 2:** Treatment timeline.

RT Mode	Treatment fractions										
SCART(VMAT)	1	2	3								
SBRT(VMAT)				1	2	3	4	5			
LRT(VMAT)									1	2	3
Date	Apr. 24	Apr. 25	Apr. 28	Apr. 29	Apr. 30	May 1	May 2	May 5	Jun.11	Jun.12	Jun.13

After the two courses of radiotherapy, systemic treatment was resumed on July 10, 2025. As of November 12, “paclitaxel liposome + carboplatin + bevacizumab” regimen was continued for 6 cycles. As of November 2025, disease remained SD (**Table 3**).

**Table 3:** Treatment timeline.

Date	Treatment phase	Key interventions
2025-02-06	CT scan after pain of right Buttocks	Tumor volume is about 399cc
2025-02-10	Recurrence confirmed after biopsy	Patient presentation with ovarian cancer sacral metastasis
2025-02–13 to 2025-04-01	Chemotherapy combined with targeted therapy	Albumin-bound paclitaxel carboplatin + bevacizumab x 3 cycles
2025-04-24→2025-05-05	SCART 3 fractions+ SBRT 5fractions	STV 54Gy/8Gy/3F PGTV 15Gy/5Gy/3F + SBRT PGTV25G/5Gy/5F
2025-06-11→2025-06-13	Lattice RT 3 fractions	VTV 45Gy/15Gy/3F, PGTV 9Gy/3Gy/3F
2025-07-10→2025-11-12	Chemotherapy combined with targeted therapy	Paclitaxet Hoosome + carboplatin + bevacizumab x 6 cycles

### Treatment planning

VMAT plans (Eclipse v16.1, AAA) were used. STV volume: 13.88cc, STV/GTV=3%. VTV volume: 1.23cc, VTV/GTV=0.4%. Radiotherapy was administered once daily with IGRT before each treatment session. STV and VTV dose distributions are shown in the figure below.

### Prescription and planning

Target Delineation and Localization: Simulation CT was fused with previous imaging (CT/MRI). GTV included the right iliac bone destruction area and invasive soft tissue components; PGTV = GTV with 3mm margin expansion.

Biological Dose (α/β=10):

Lattice VTV BED10 ≈ 112.5 Gy; EQD2 ≈ 93.8 Gy.

SCART STV BED10 ≈ 151.2 Gy; EQD2 ≈ 126 Gy.

SBRT(25Gy/5f) PTV BED10 ≈ 37.5 Gy; EQD2 ≈31.25Gy.

## Results

Treatment efficacy was evaluated according to RECIST 1.1 criteria:

Baseline evaluation: Right iliac giant bone metastatic lesion approximately 89×78×58mm, calculated volume approximately 399.0cc, estimated SLD: 9.13 cm (**Figure 1a**).

Post-SCART(1 month): Lesion volume evaluated as 308cc, estimated volume change: -22.8%, SLD: 8.38 cm (change: -8.3%), efficacy evaluation: SD (stable disease) (**Figure 1b**).

Post-Lattice (6 months): Follow-up MRI showed tumor volume reduced to 136.1cc, GTV: 136.1 cc (change: -65.9%), estimated SLD: 6.38 cm (change: -30.1%), efficacy evaluation: PR (partial response) (**Figures 1c, d**).

Throughout the treatment process, patient PS score was 0-1, indicating good performance status and ability to tolerate subsequent treatment. NRS score decreased from pre-treatment 4–5 to 2.

## Discussion

This case presents successful treatment of giant pelvic bone metastasis using sequential spatial fractionation techniques. Initial SCART therapy provided significant pain relief but insufficient tumor shrinkage, prompting Lattice dose-escalation that achieved substantial tumor regression. The sequential approach proved effective for this radiotherapy-resistant metastasis.

Clinically, 2% of ovarian cancer patients are diagnosed with bone metastasis (6). However, actual bone metastasis incidence is higher than this data. According to autopsy data, actual bone metastasis incidence in ovarian cancer is approximately 10% (7), with most common bone metastasis sites being lumbar spine and pelvis (8). Studies show that late clinical stage, poor differentiation, and lymph node metastasis are associated with the development of bone metastasis in ovarian cancer (9). The relationship between ovarian cancer histological type and bone metastasis remains controversial, with some studies suggesting ovarian cancer histological type is unrelated to bone metastasis occurrence and development (10). However, other studies suggest ovarian clear cell carcinoma patients are more prone to bone metastasis (11), and this case’s pathological type and bone metastasis characteristics are consistent with these patterns.

**Figure 2:**
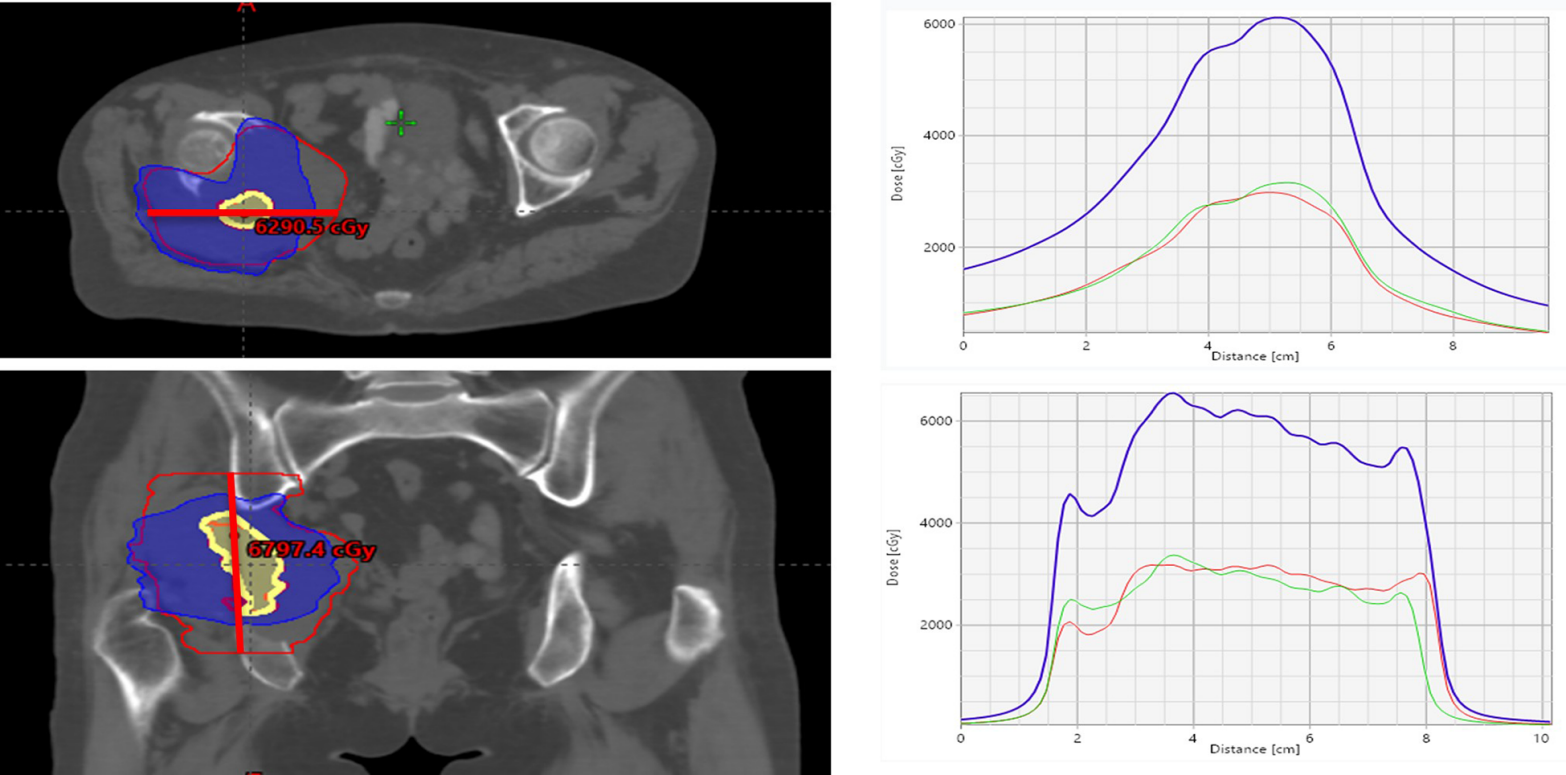
SCART plan. CT scan images display colored radiation dose regions with peak levels of 6290.5 cGy and 6797.4 cGy. The comprehensive four-panel visualization includes: upper left showing axial plane dose distribution, upper right displaying peak-valley dose curves through vertices, lower left presenting coronal plane distribution, and lower right illustrating additional peak-valley dose distribution curves. Graphs on the right show dose distribution patterns plotted against distance in centimeters, providing detailed analysis of radiation dose patterns across different anatomical planes and treatment configurations for comprehensive radiation therapy assessment.

SCART technique proposed by Dr. Yang Jun, as an emerging precision radiotherapy technology, has advantages of highly conformal dose distribution and small normal tissue damage (12, 13) (**Figure 2**). In this case, we first used SCART combined with SBRT technique for treatment. One month after treatment, patient’s pain symptoms were significantly alleviated, but imaging examination showed tumor volume reduced from initial volume to 308 cc, with magnitude of reduction did not meet PR criteria, suggesting potential insensitivity of the lesion to initial radiotherapy.

**Figure 3:**
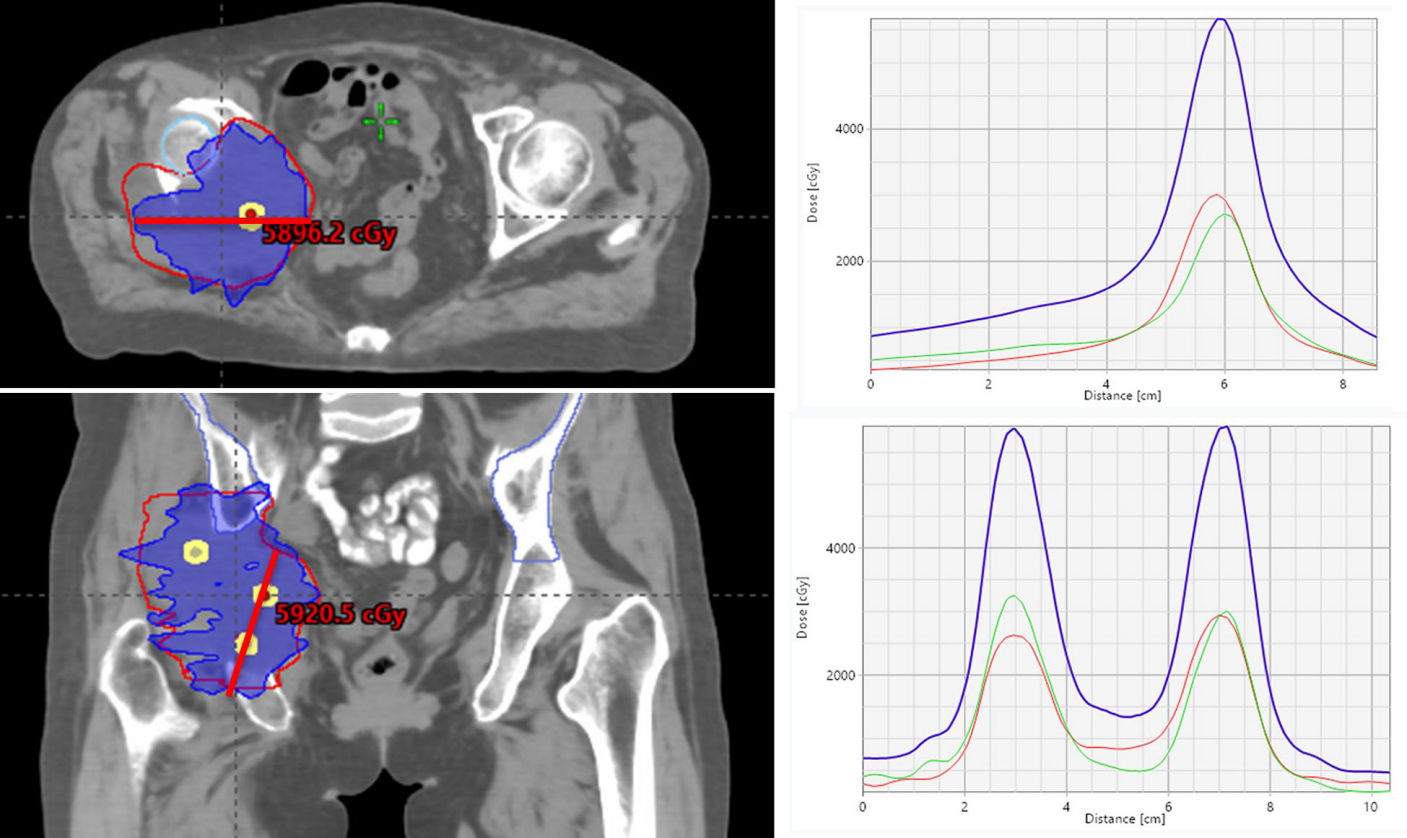
LRT plan. CT scans display radiation dose distributions across anatomical planes using colored contour lines measured in centigray. Four-panel visualization shows: upper left axial plane distribution, upper right peak-valley edge versus hotspot patterns, lower left coronal plane mapping, and lower right peak-valley curves through dose vertices. Graphs illustrate dose intensity variations with blue, red, and green curves representing different dose peaks. Dose distribution curves demonstrate single field arcs (red-green) and combined arcs (blue). Lattice radiation therapy exhibits peak-to-valley ratios of approximately 3:1, indicating significant dose modulation for targeted treatment optimization.

For bulky tumor lesions, especially those with poor response to conventional radiotherapy, Lattice technique provides an effective therapeutic strategy (14). Lattice technique, as an important advancement in spatial fractionated radiotherapy, creates dose peak areas within the tumor, forming a “lattice-like” dose distribution that can increase irradiation dose to cold spot areas while ensuring safe dose at target margins. Wu et al. (15) demonstrated that Lattice technique can effectively improve local control rate of large-volume tumors while reducing surrounding normal tissue damage (**Figure 3**).

In this case, after using Lattice technique for dose-escalation radiotherapy, tumor volume further reduced to 136.1cc at 6-month follow-up. The combination of two spatial fractionation techniques promoted tumor regression in radiotherapy-resistant tumor types without increasing Grade ≥3 acute adverse events. The pain relief after the first course is rapidly reduction, While imaging changes may not be apparent early after high-dose radiotherapy. But 1-month follow-up enables to assessment of acute toxicity reactions and establishment of baseline data for subsequent treatment decisions.

**Figure 4:**
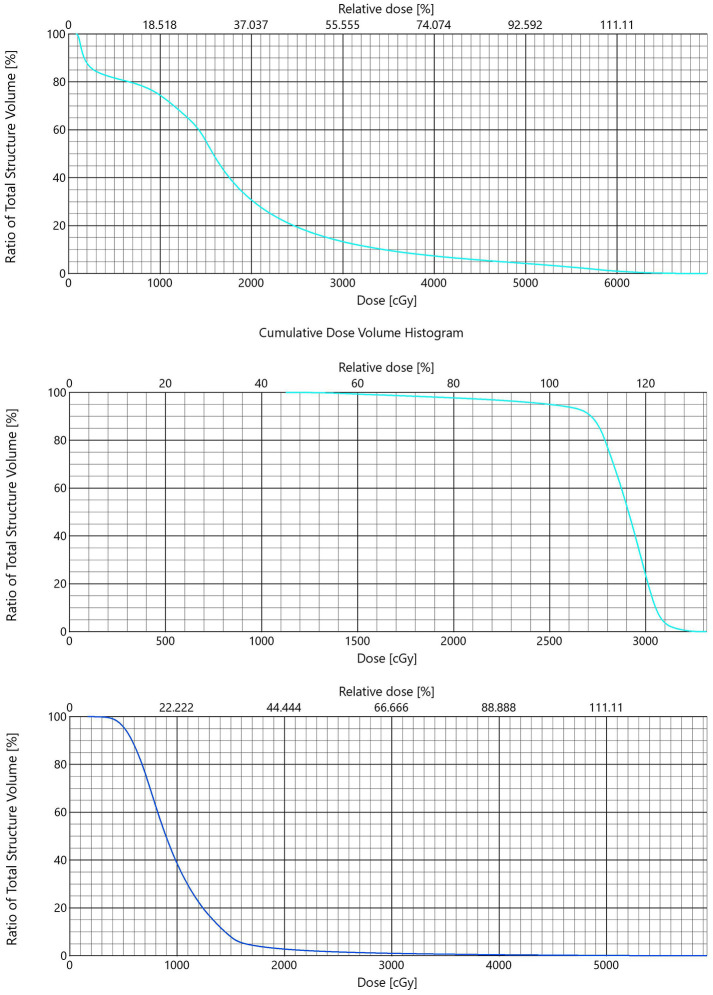
DVH of SCART, SBRT and Lattice. Three dose volume histograms are displayed in a vertical sequence. The first graph shows data labeled “SCART” with a descending curve from 100% to near 0% along the dose axis up to about 6000 cGy. The second graph, labeled “SBRT,” depicts a plateau before a rapid decline from 100% to near 0% over the dose range of about 0 to 3000 cGy. The third graph, labeled “LRT” also shows a curve starting at 100% and descending smoothly towards 0% along the dose axis up to about 5500 cGy.

SCART represents a significant technological advancement beyond conventional SBRT, primarily distinguished by its unique capability to achieve peak-to-valley ratios exceeding 300% of the peripheral dose—a feat unattainable with standard SBRT hotspots, which typically cannot exceed 150-200% of peripheral dose. This distinctive dose distribution characteristic is not merely a result of “removing limits” but rather reflects an innovative technology with sophisticated dose sculpting capabilities and enhanced biological effects (**Figure 4a**).

The therapeutic synergy between SCART and Lattice techniques stems from their complementary spatial dose distribution patterns. While SCART employs centralized focusing to achieve maximum dose concentration in the tumor core, Lattice radiotherapy creates multiple high-dose “islands” distributed throughout the tumor volume through spatial fractionation. This complementary approach enables comprehensive tumor coverage, with Lattice particularly effective in targeting multiple tumor regions, including potential residual peripheral areas that may receive suboptimal dose from central focusing alone. The sequential application thus provides both intensive central tumor ablation and comprehensive peripheral coverage (**Figure 4c**).

SCART and Lattice techniques offer distinct advantages over conventional radiotherapy approaches across multiple dimensions:

1. Precision Targeting and Dose Optimization: Highly conformal dose distribution ensures accurate tumor coverage while maximizing normal tissue sparing. Advanced dose optimization eliminates hotspots and coldspots, ensuring uniform tumor coverage. Particularly advantageous for pelvic lesions where critical structure protection is essential.

2. Treatment Efficiency and Safet.

Shorter treatment courses without delaying systemic therapy initiation. Demonstrated efficacy in less sensitive large-volume tumors when surrounding tissue safety is maintained.

3. Technological Superiority: Compared with existing radiotherapy modalities (**Figure 4b**):

- vs Conventional SBRT: Higher central dose delivery with enhanced peripheral protection
- vs Traditional Lattice: More precise dose control and stronger biological effects
- vs Proton Therapy: Better cost-effectiveness and broader clinical applicability

This combined spatial fractionation approach provides new treatment options for bulky tumors that have historically presented therapeutic challenges. The sequential SCART and Lattice application strategy establishes a new paradigm for managing large-volume malignancies, offering a balance between aggressive tumor control and treatment tolerability. This approach warrants further investigation in prospective clinical trials to establish standardized protocols and validate efficacy across broader patient populations.

### Limitations and Future Directions

The technique requires advanced equipment and technical expertise. Long-term efficacy and safety need validation through larger studies. The immunomodulatory effects of spatial fractionated radiotherapy warrant further investigation (16, 17).

## Conclusion

Sequential SCART followed by Lattice radiotherapy appears safe, feasible, and effective for managing bulky bone metastases in ovarian cancer. SCART incorporates tumor heterogeneity and regional variations in radiosensitivity into its treatment planning, with sequential optimization strategies employed to maximize therapeutic efficacy. Significant tumor regression, symptomatic improvement, and tolerable toxicity were observed. Larger prospective studies are needed to determine long-term outcomes and identify ideal treatment candidates.

## Data Availability

The original contributions presented in the study are included in the article/supplementary material. Further inquiries can be directed to the corresponding author.
